# Interplay of Modified Sialic Acid Inhibitors and the Human Parainfluenza Virus 1 Hemagglutinin-Neuraminidase Active Site

**DOI:** 10.1021/acsmedchemlett.3c00291

**Published:** 2023-09-26

**Authors:** Paola Rota, Paolo La Rocca, Francesco Bonfante, Matteo Pagliari, Federica Cirillo, Marco Piccoli, Andrea Ghiroldi, Valentina Franco, Carlo Pappone, Pietro Allevi, Luigi Anastasia

**Affiliations:** † Department of Biomedical, Surgical and Dental Sciences, Università degli Studi di Milano, 20133 Milan, Italy; ° Institute for Molecular and Translational Cardiology, San Donato Milanese, 20097 Milan, Italy; ⊗ Department of Biomedical Sciences for Health, Università degli Studi di Milano, 20133 Milan, Italy; § Division of Comparative Biomedical Sciences, Istituto Zooprofilattico Sperimentale delle Venezie, 35020 Legnaro, Italy; ∥ Laboratory of Stem Cells for Tissue Engineering, IRCCS Policlinico San Donato, San Donato Milanese, 20097 Milan Italy; + Division of Clinical and Experimental Pharmacology, Department of Internal Medicine and Therapeutics, University of Pavia, 27100 Pavia, Italy; © IRCCS, Mondino Foundation, 27100 Pavia, Italy; & Arrhythmology Department, IRCCS Policlinico San Donato, Piazza Malan 2, San Donato Milanese, 20097 Milan Italy; # Faculty of Medicine, University of Vita-Salute San Raffaele, 20132 Milan, Italy; ∇ Division of Pediatric Infectious Diseases, Department for Women’s and Children’s Health, University of Padua, 35128 Padua, Italy

**Keywords:** human parainfluenza virus 1, sialic acid, antiviral inhibitor, hemagglutinin-neuraminidase, viral infection

## Abstract

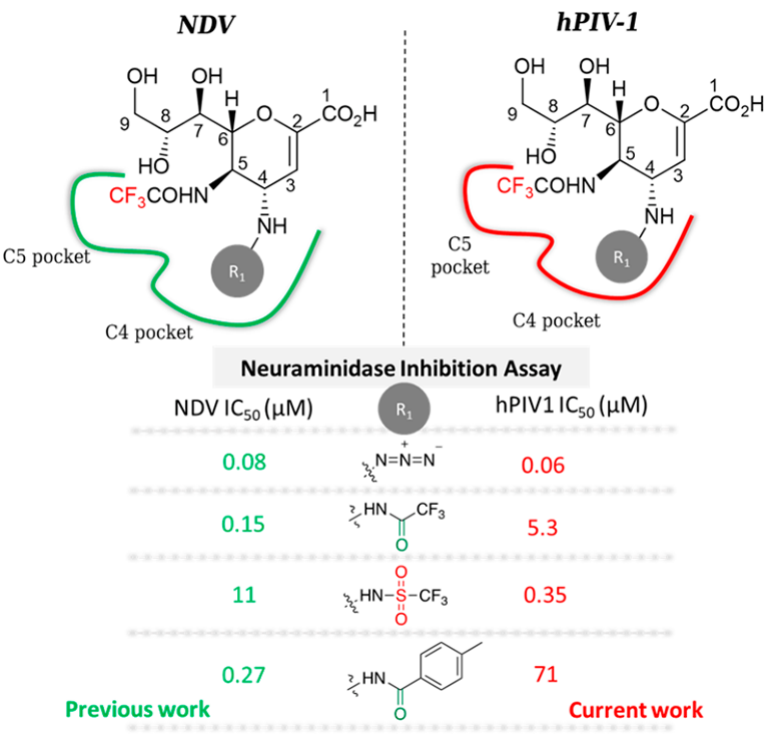

In the search for effective antivirals against Paramyxoviridae, the dynamics of human parainfluenza virus type 1 hemagglutinin-neuraminidase (hPIV1-HN) inhibition offers a promising perspective. This study focuses on the potential of C5- and C4-modified 2,3-unsaturated sialic acid (DANA) inhibitors and highlights their interaction with the hPIV1-HN enzyme. We show that a strategic substitution, replacing the C5 isopropyl group in BCX 2798 with a trifluoroacetyl function, increases inhibitory potency 3- to 4-fold. At the same time, we explore the special properties of the catalytic site of hPIV1-HN, which harbors only small substituents and favors a C4 sulfonylamido function over a carbonyl function, in contrast to the C4 pocket of Newcastle disease virus hemagglutinin-neuraminidase (NDV-HN). Based on these findings, we present a newly identified potent inhibitor that has the preferred C5 trifluoroacetamido and C4 trifluorosulfonylamide groups. The results of this study pave the way for a deeper understanding of the C4 and C5 binding pockets of hPIV1-HN and promote the development of new, more selective inhibitors.

Human parainfluenza virus types 1–3 (hPIV 1–3) are usually self-limiting pathogens, analogous to influenza A and B viruses. Occasionally, however, they cause a wide range of respiratory symptoms leading to hospitalization, mainly due to bronchiolitis and pneumonia.^1−4^ While seasonal vaccines and medications are available against influenza A and B viruses,^2−4^ there are no approved antiviral agents or vaccines against hPIVs to date.

Significant results in this area involve the synthesis of potent 2,3-unsaturated sialic acid derivatives (DANA, Neu5Ac2en, **1**) targeting viral neuraminidase (N), such as the commercially available Zanamivir **2**, or hemagglutinin neuraminidase (HN), such as the agent BCX-2798 directed against hPIV1 **3** (Figure 1).^4,5^ Nonetheless, developing effective antiviral therapies for hPIVs remains a critical objective.

**Figure 1:**
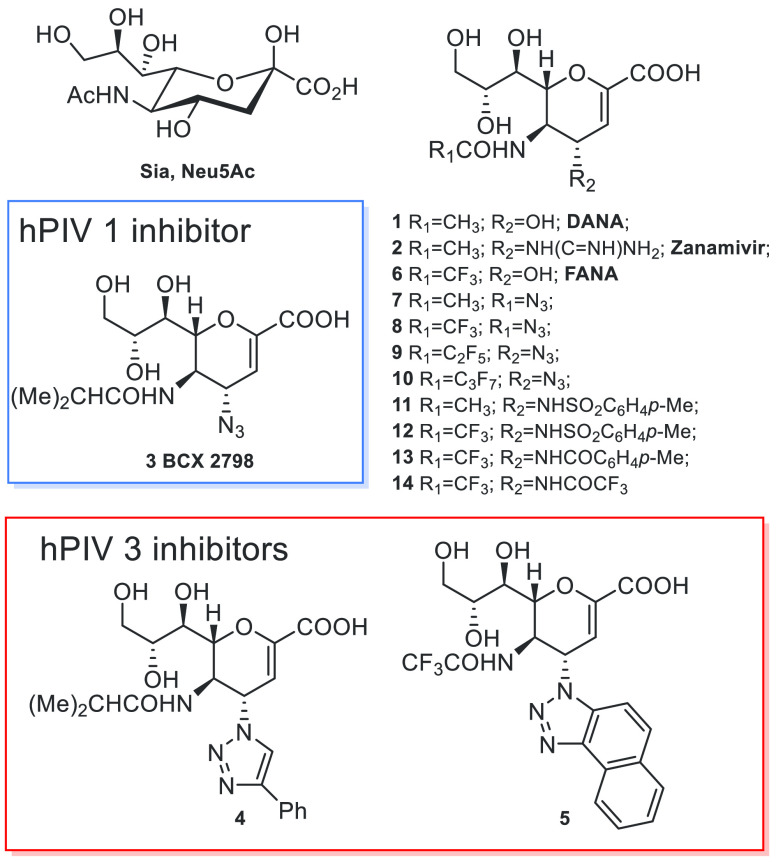
Inhibitors derived from 2,3-unsaturated sialic acid.

The hPIVs HN is an ideal drug target because it serves multiple regulatory functions at different stages of the hPIV life cycle: (i) cell adhesion, (ii) virus release, and (iii) fusion process.^1,5^ In particular, the presence of crystallographic structures^6,7^ of both Newcastle disease virus hemagglutinin-neuraminidase (NDV-HN) and hPIV3-HN favors the development of inhibitors for these targets; otherwise, the lack of a 3D structure for hPIV1-HN makes the pathway complex. Despite the homology model^8^ developed using hPIV3 as template for predicting inhibitors of hPIV1, the most active agents available to date are BCX 2798, which is based on the crystallographic structure of NDV-HN (NI IC_50_ between 0.04 and 0.5 μM and HI IC_50_ between 0.08 and 0.124 μM), and its saturated 2α,3β-difluoro derivative (NI IC_50_ = 0.09 μM and HI IC_50_ = 2.14 μM ).^5,8,9^

Moreover, the C4 modified phenyltriazolic derivative of BCX 2798, compound **4**, and the naphtyltriazolic compound **5**, derived from FANA **6**, showed very low IC_50_ values (NI 2.7 and 0.57 μM) on hPIV3-HN.^10,11^ Otherwise, the C3 *N*-linked inhibitor **4** showed higher IC_50_ value on hPIV1-HN (NI 21.88 μM), while no data for compound **5** on hPIV1-HN have been reported.^12^

Although some interesting results have been obtained with these hPIVs-HN inhibitors in *in vitro* studies, the current treatment of choice against hPIVs combines ribavirin with corticosteroids and/or epinephrine. Alternatively, a host-directed therapy (DAS181) based on a recombinant sialidase protein is under investigation (clinical trials, phase 3).^13,14^ Therefore, new compounds and their biological and virological evaluations are constantly needed in this field.

Recently, in a study aimed at using NDV-HN as the best predictive model for the development of hPIV1 inhibitors,^15−19^ we found very potent inhibitors of NDV-HN: the C4 azido compounds **7**–**10**, the *p*-toluenesulfonamido derivatives **11** and **12**, the *p*-tolueneamido derivative **13** and the trifluoroacetamido one **14** (Figure 2). All these compounds, except the azido derivative **9**, showed high neuraminidase inhibitory activity in the low nanomolar range (IC_50_ values ranging from 0.05 to 0.50 μM, see Supporting Information, Table S1).

**Figure 2:**
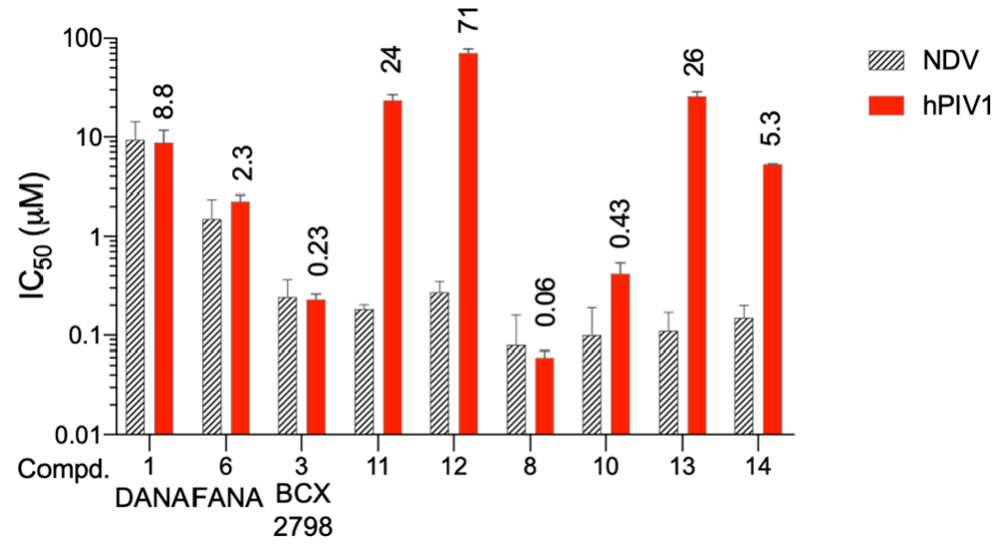
Neuraminidase inhibition assay. IC_50_ values for DANA **1**, FANA **5**, BCX 2798 **3**, and some analogs modified at the C4 and C5 positions on hPIV1-HN (red), compared with values previously obtained on NDV-HN^19^ (gray stripes, values shown are the mean of IC_50_ values obtained on three NDV strains; see also Supporting Information). In the hPIV1-HN assay, each value represents the mean of three independent experiments performed in triplicate.

These molecules have also been shown to be highly effective inhibitors of viral replication, as we have demonstrated that they prevent the release of viral particles from infected cells.^19^

Encouraged by the results obtained and stimulated by the interest in biologically active sialic acid derivatives,^20−24^ we investigated the inhibitory activity against hPIV1-HN of the compounds previously selected as very potent against NDV-HN. Specifically, we performed the neuraminidase inhibition (NI) assay on *in-toto* inactivated hPIV1 using the fluorogenic neuraminidase substrate 2′-(4-methylumbelliferyl)-α-d-*N*-acetylneuraminic acid (4-MUNeu5Ac).

We chose the following as reference compounds: DANA **1** and FANA **6** as nonselective sialidase inhibitors^15,16^ and BCX2798 **3**(5,8) as the most potent and studied inhibitor against hPIV1. Furthermore, we selected the best NDV-HN inhibitors we found:^19^ the *p*-toluenesulfonyl derivatives **11** and **12**, the more promising 4-azido C5-fluorinated amides **8** and **10**, the C4 *p*-tolyl analog **13**, and the C4 trifluoroacetamido compound **14** (see Supporting Information, Table S1).

DANA **1** and FANA **6** showed IC_50_ values in the micromolar range (8.8 and 2.3 μM, respectively), consistent with those of NDV-HN. Interestingly, the IC_50_ value of DANA for hPIV1-HN is well known in the literature,^8^ whereas that of FANA is, to our knowledge, reported here for the first time, supporting the idea that the fluorine atoms at C5 could potentially enhance the interactions also in hPIV1-HN. Moreover, the benchmark inhibitor BCX 2798 showed an IC_50_ value (0.23 μM) against hPIV1-HN in agreement with the literature (IC_50_ = 0.04–0.50 μM)^5,8,9^ and comparable to the value observed against the three NDV-HN strains (IC_50_ between 0.11 and 0.32 μM).^19^

A major novelty is the analysis of the selected compounds *p*-toluenesulfonyl derivatives **11** and **12** and C4 *p*-tolyl analog **13**, which showed a worse neuraminidase inhibitory activity on hPIV1 (IC_50_ between 24 and 71 μM) than that observed for NDV (IC_50_ between 0.18 and 0.30 μM). On the other hand, the result obtained with C5-fluorinated azides **8** and **10** (IC_50_ = 0.06 and 0.43 μM) is impressive, although not surprising, considering the results obtained for NDV-HN. Overall, these results led us to hypothesize that the hydrophobic C4 pocket of the catalytic site of hPIV1-HN has a new unprocessed feature. We confirmed that it can accommodate small groups such as azides. However, unlike NDV, it is not large enough to accommodate bulky groups such as *p*-toluenesulfonyl and *p*-tolylamides.

Interestingly, the small C4 trifluoroacetamido compound **14** showed discrete hPIV1 inhibitory neuraminidase activity (5.3 μM) in the micromolar range, albeit significantly higher than that obtained for NDV-HN (0.15 μM), indicating that the trifluoroacetamido moiety is not sufficiently capable of drastically enhancing the interactions with the C4 catalytic pocket of hPIV1.

Overall, the data obtained from this initial screen enabled the discovery of a new inhibitor of hPIV1 neuraminidase activity, C5 trifluoroacetamido azido derivative **8**, which is 3–4 times more active than BCX 2798.

These new results complement previous findings on the C5 pocket by El-Deeb et al.^8^ and highlight the importance of modifications in this position, as we have previously reported.^25^ So, we can partially conclude that, by conducting *in vitro* experiments involving both NDV-HN and hPIV1-HN, we have found that relying solely on molecular modeling and simulations with NDV-HN does not produce accurate predictions on hPIV1-HN.

In view of the results described above and the low predictability of the 3D model used, we decided to select and test additional C4 sulfonamides **15**–**20** and amides **21**–**23** to better understand the properties of the C4 hPIV1 HN-binding domain (Figure 3 and Supporting Information, Figure S1). The IC_50_ values of hPIV1 HN for all these molecules have not yet been reported.

**Figure 3:**
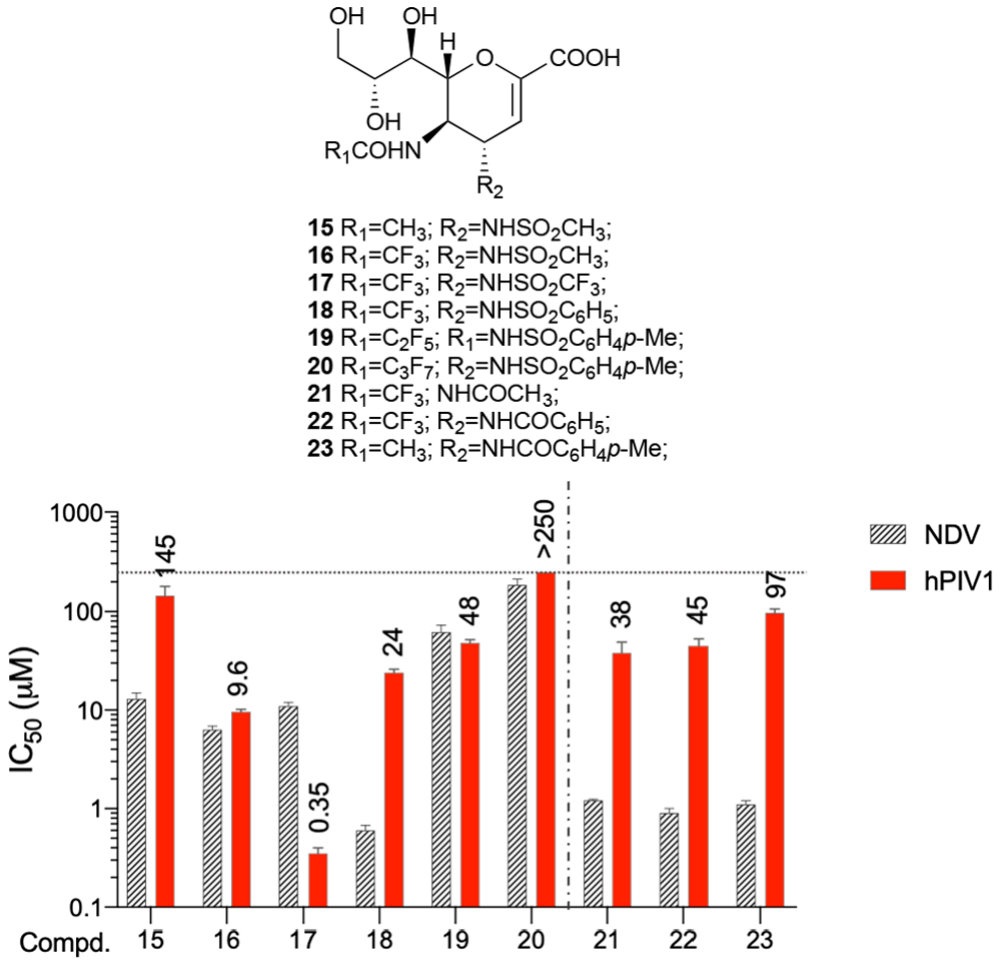
Neuraminidase inhibition assay and chemical structures of compounds **15**–**23**. IC_50_ values of some analogs modified at the C4 and C5 positions on hPIV1-HN and comparison with those previously obtained on NDV-HN.^19^ In the hPIV1-HN assay, each value represents the mean of three independent experiments performed in triplicate.

First, we considered compounds **19** and **20** with a hindered C4 group coupled to a specific fluorinated C5 chain. We confirmed that bulky substituents at both C4 and C5 fit poorly into the catalytic pocket of hPIV1-HN (IC_50_ = 48 and >250 μM, respectively), as observed for NDV (IC_50_ = 62 and 186 μM, respectively) and unlike hPIV3 (well evidenced by literature compounds **4** and **5**).^10,11^ These data confirm that the active site of hPIV3-HN appears to be different from that of hPIV1-HN, at least in the pocket region relative to the C4 position. This evidence is also confirmed by the results for compounds **18**, **22**, and **23** (IC_50_ on NDV HN = 0.6, 0.9, and 1.1 μM, respectively).

On the other hand, IC_50_ values remained in the micromolar range (9.6–145 μM) when a small methanesulfonamide at position 4 with a C5 normal or trifluoroacetamido substituent was retained (compounds **15** and **16**, respectively). Remarkably, the presence of a C5 trifluoroacetamido group makes the compound significantly more active than the corresponding acetamide, as previously observed with NDV.

Interestingly, we were also able to make some assumptions about the role of the sulfonyl and carbonyl groups and their influence on interactions with the C4 binding pocket of hPIV1. Specifically, replacing the sulfonyl group of **16** with the carbonyl group to compound **21** resulted in a significant decrease in activity with a higher IC_50_ (38 μM).

This evidence also holds when large substituents are considered, as observed for compounds **18** and **22** (with a phenyl substituent on the C4 sulfonyl and carbonyl groups, respectively) and compound **23** (with a C4-*p*-toluoylamide), the analog of C4 sulfonamide compound **11**. In contrast to what we observed for NDV-HN, the interactions between the C4 substituent and the corresponding binding pocket of the catalytic site of hPIV1-HN appeared to be enhanced by the presence of a sulfonyl amide group instead of the carbonyl group.

Consistent with this observation, we decided to investigate compound **17**, a structural analog of compound **14** but containing a trifluorosulfonamide instead of a trifluoroacylamido group. Surprisingly, compound **17** showed significantly high inhibitory activity with an IC_50_ in the low nanomolar range (0.35 μM), definitely confirming that the trifluoromethyl group at C4 instead of a methyl group seems to enhance the interactions with the catalytic site of both NDV-HN and hPIV1-HN. However, in the case of hPIV1-HN, this positive effect is exclusively associated with the simultaneous presence of the sulfonyl group (see Figure S1).

This SAR study provided a better understanding of the C4/C5 hPIV1 active site pocket since no crystal structure is currently available. We demonstrated that the catalytic center of hPIV1 shares only some specific features (such as the C5 binding pocket) with those of NDV and hPIV3 but that there are some substantial differences, especially with respect to the C4 binding pocket.

Since the best neuraminidase inhibitor of hPIV1-HN discovered in this study was compound **8**, which combines a C5 trifluoroamide with a C4 azide, we decided to test two other members of the azido family: compound **7**, which has an acetylamide at C5, and the pentafluoropropanamide derivative **9**, which is an intermediate chain between that of compound **8** and that of **10**. Interestingly, we observed the same IC_50_ trend for these molecules and the C4 hydroxy analogs on NDV-HN that we reported previously.^15,16^

The neuraminidase IC_50_ values of compounds **7** and **9** (0.46 and 3.0 μM, respectively, see Table S1) confirmed the azido group as the ideal C4 substituent. Notably, substitution of the trifluoroacetamido group present in inhibitor **8** by the acetamido group of compound **7** decreased the inhibitory activity, although it remained in the micromolar range. In addition, during the transition from the trifluoroacetamido to the corresponding *N*-pentafluoropropanamide of compound **9**, we observed an increase in the IC_50_ value, which is even higher than that of *N*-heptafluorobutyramide **10** (0.42 μM).

Because HN is a multifunctional protein,^1,7^ it was also important to evaluate the ability of these promising molecules to inhibit hPIV1-HN hemagglutination function (HI). Indeed, whereas an anti-hemagglutinin effect of these potent inhibitors is not observed in NDV because of the presence of a second (inhibitor-insensitive) sialic acid-binding site on the HN surface,^26,27^ this second site remained hidden in hPIVs.^28^

Therefore, we investigated the ability of azido derivatives to inhibit hPIV1 HN by performing a hemagglutinin inhibition assay (HI). As shown in Figure 4, both reference compounds, DANA and BCX 2798, had IC_50_ values consistent with those reported in the literature (>10 μM, threshold chosen in this study and 0.10 μM, respectively).

**Figure 4:**
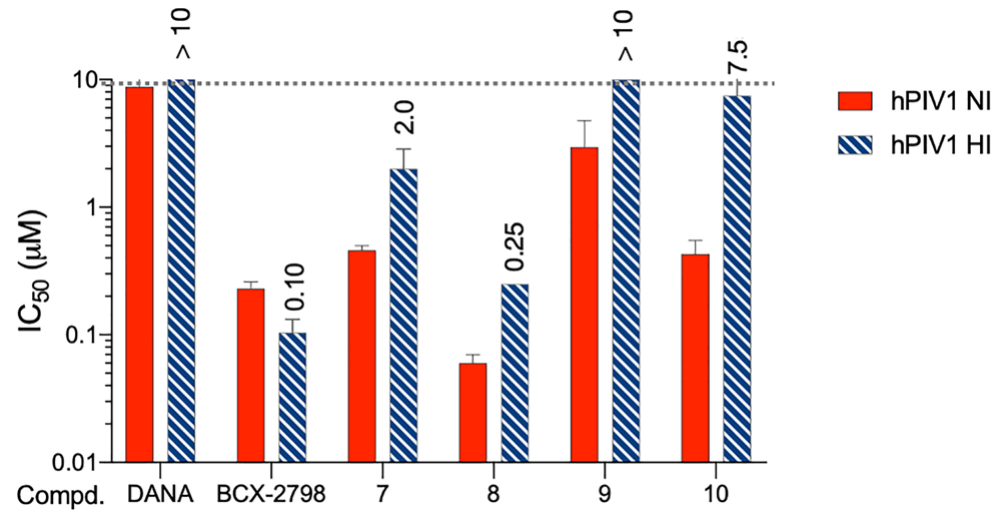
Hemagglutination and neuraminidase inhibition assays using hPIV1 (Sendai strain) to evaluate DANA, BCX 2798, and compounds **8**–**10** (azido series). We have plotted in the graph the mean values of IC_50_ values from the neuraminidase inhibition assay performed with three different NDV strains (red) and the IC_50_ values from the hemagglutinin inhibition assay (blue striped). The IC_50_ value is the mean of three independent experiments performed in triplicate.

Remarkably, the azido series showed an IC_50_ value trend very similar to that observed in the NI assay. Compound **7** showed an IC_50_ value of 2.0 μM, and its trifluorinated analog **8** showed an 8-fold lower value of 0.25 μM. The elongation of the fluorinated chain leads to a decrease of the inhibitory effect in compound **9** (IC_50_ > 10 μM), which is partially restored in compound **10** (IC_50_ = 7.5 μM).

In conclusion, we found a very potent hPIV1 inhibitor in compound **8**, whose neuraminidase inhibitory activity is 3–4 times stronger than that of the current reference molecule BCX-2798. Moreover, this molecule also inhibits hPIV hemagglutinin activity in the nanomolar range. This result suggests that the presence of short fluorinated amides at C5 interacts with the C5 active site pocket of hPIV 1 as well as with the isopropyl group of BCX 2798.

Furthermore, our study revealed notable distinctions between our findings for NDV-HN and the previously reported literature findings for hPIV3^10,29,30^ regarding the C4 pocket of the catalytic site of hPIV1-HN. Specifically, we observed that the C4 pocket of hPIV1-HN is primarily suited for accommodating relatively small substituents. Additionally, we found that a C4 sulfonylamidic group is preferred over a carbonyl group in this context. Building upon these observations, we made a significant discovery of a novel C4 trifluoromethanesulfonamido substituent that exhibits exceptional potency with an IC_50_ value in the nanomolar range. The enhanced activity resulting from the addition of fluorine atoms at the C4 and C5 positions could be attributed to their polar hydrophobic nature,^15^ which enables the formation of weak hydrogen bonds. These findings offer novel insights into the replacement of the Neu5Ac2en C4 and C5 functionalities of hPIV1-HN, thereby expanding our understanding of its catalytic site and furthering our existing knowledge in this field. Moreover, the trifluoromethanesulfonamide at C4, as an alternative to the azido group, could represent the starting point for future studies.

## Experimental Section

No unexpected or unusually high safety hazards were encountered.

### Chemicals

All chemicals and solvents used were of analytical grade and were purchased from Sigma-Aldrich (Merck). Deionized water was prepared by filtering water on a Milli-Q Simplicity 185 filtration system from Millipore (Merck). All the molecules have been synthesized according to the recent literature.^19^ All the tested compounds showed a purity >95%.

### Viruses

NDV La Sota “Clone 30” was grown and purified as described previously in the literature,^31^ La Sota 40/14 (inactivated) and the velogenic (inactivated) strain APMV-1/chicken/Egypt/13VIR-5009-2/2013) were obtained from Istituto Zooprofilattico Sperimentale delle Venezie. hPIV1 inactivated virus (Sendai strain) was purchased from HyTest Ltd. Stock viruses were harvested, titrated, and stored at −80 °C until use.

### Neuraminidase Activity Inhibition Assay on hPIV1-HN

Neuraminidase activity inhibition (NI) assay was performed by using 4-MUNeu5Ac as the artificial substrate. Briefly, the incubation mixture (final volume of 100 μL) contained 0.2 μg of hPIV1, and different amounts of the inhibitors (0–2.0 mM), 0.12 mM 4-MU-Neu5Ac, 600 μg of bovine serum albumin (BSA), and acetate buffer (NaOAc 50 mM, CaCl_2_ 5 mM), pH 5.0, were incubated. After incubation at 37 °C for 30 min, the reactions were stopped by the addition of 1.5 mL of 0.2 M glycine buffered with NaOH at pH 10.8, and the neuraminidase activity was determined by spectrofluorometric measurement (Varioskan LUX Multimode Microplate reader, Thermo Fisher Scientific) of the 4-methylumbelliferone released (λ excitation 365 nm, λ emission 448 nm). 1 mU of neuraminidase activity of 1 mg/min is defined as the amount of enzyme releasing 1 nmol of *N*-acetylneuraminic acid per minute at 37 °C. Seven concentrations of each inhibitor were used to determine the IC_50_ with a fixed concentration (0.12 mM) of 4-MUNeu5Ac. The IC_50_ values are the means of three independent experiments performed in triplicate.

### Hemagglutinin Activity Inhibition Assay

Hemagglutinin activity inhibition (NI) assay was performed using chicken red blood cells (C-RBC, obtained from Istituto Zooprofilattico Sperimentale delle Venezie), according to El-Deeb et al.^8^ Compounds were diluted in PBS as a 3× solution for each concentration tested (25 μL/well, 1× final). Each dilution was mixed with 4 hemagglutination units (HAU) of inactivated hPIV1 (25 μL/well, 1 HAU final) and incubated for 20 min at room temperature. The plate was transferred on ice, and then an equivalent volume of ice-cold 1% C-RBC (25 μL/well) was added to each well. Plate was incubated for 1 h and 30 min at room temperature before reading the hemagglutination results. Noteworthy, the HN inhibitors were assessed in triplicate in V-bottom 96-well plates. The IC_50_ value for HI assays was defined as the compound concentration that gives similar agglutination to that observed in a well containing only 0.5 HAU of hPIV1 and C-RBC.

### Statistical Analysis

Statistical analysis was performed using GraphPad Prism version 8.0 (GraphPad Software, San Diego, CA, USA). The data are presented as means ± standard deviation (SD) from a minimum of two experiments conducted in triplicate. The IC_50_ values for the various assays were determined by employing nonlinear regression curve fitting, specifically the inhibitor versus normalized response with a variable slope equation, by using GraphPad Prism 8.0 software.

## Supporting Information Available

IC_50_ values for all the final compounds and 3D representations of some important compounds (PDF)

## Abbreviations

hPIV: human parainfluenza virus
NDV: Newcastle disease virus
HN: hemagglutinin-neuraminidase
IC_50_: half-maximal inhibitory concentration
DANA or Neu5Ac2en: *N*-acetyl-2,3-dehydro-2-deoxyneuraminic acid
N: neuraminidase
4-MUNeu5Ac: 2′-(4-methylumbelliferyl)-α-d-*N*-acetylneuraminic acid
NI: neuraminidase inhibition
FANA: *N*-trifluoroacetyl-2,3-dehydro-2-deoxyneuraminic acid
HI: hemagglutinin inhibition
SAR: structure–activity relationship
